# Clinical characteristic of isolated thrombocytopenia in patients with bone marrow failure-related germline variants: a retrospective study from a single centre

**DOI:** 10.1080/07853890.2025.2523560

**Published:** 2025-06-26

**Authors:** Nanxi Dong, Jingjie Dong, Chuanao Xin, Peicheng Wang, Yaonan Hong, Rudan Zheng, Zexing Sun, Qi Liu, Yingying Shen, Xiawan Yang, Yiping Shen, Jianping Shen, Baodong Ye, Yuhong Zhou, Dijiong Wu

**Affiliations:** a Department of Hematology, The First Affiliated Hospital of Zhejiang Chinese Medical University (Zhejiang Provincial Hospital of Chinese Medicine), Hangzhou, Zhejiang, China; b The First School of Clinical Medicine, Zhejiang Chinese Medical University, Hangzhou, Zhejiang, China; c National Traditional Chinese Medicine Clinical Research Base (Hematology), Hangzhou, Zhejiang, China; d Department of Oncology and Hematology, Wenzhou Hospital of Integrated Traditional Chinese and Western Medicine Affiliated to Zhejiang Chinese Medicine University, Wenzhou, Zhejiang, China

**Keywords:** Heterozygous germline variants, isolated thrombocytopenia, mean corpuscular volume, whole exome sequencing

## Abstract

**Background:**

Immune thrombocytopenia (ITP) comprises the majority of thrombocytopenia. Some patients respond poorly to first-line ITP therapy or develop pancytopenia years later. Recent studies link heterozygous germline variants in acquired aplastic anemia (AA), yet their role in isolated thrombocytopenia carrying bone marrow failure-related germline variants (ITGV-BMFs) remains unclear. While whole-exome sequencing (WES) detects these variants, its cost limits routine use. This study compares prognosis and clinical features of isolated thrombocytopenia in patients with ITGV-BMFsand those with classic ITP.

**Methods:**

The clinical data of patients diagnosed with ITGV-BMFs were retrospectively analyzed and compared with those of patients with classic ITP from August 2018 to February 2024. The baseline characteristics, genomic systematically background, previous treatment response as well as their follow-up outcomes were compared.

**Results:**

Patients with ITGV-BMFs demonstrated earlier onset age (*p* < 0.001), lower bleeding scores and CD34%, along with elevated mean corpuscular volume (MCV), mean corpuscular hemoglobin (MCH), and reticulocyte (RET) counts (*p* < 0.001). Multivariate logistic regression analysis showed that the patients with ITGV-BMFs may possess distinct characteristics, including an earlier age at onset (*p* = 0.014) and lower bleeding score (*p* = 0.048). Notably, MCV and RET showed promising performance in receiver operating characteristic (ROC) curve analysis. During the follow-up period, 57.69% (15/26) ITGV-BMFs patients were further confirmed as aplastic anemia (AA, *n* = 13) or myelodysplastic syndrome (MDS, *n* = 2), with a median progression-free survival (PFS) of 7.25 years (*p* < 0.0001).

**Conclusion:**

ITGV-BMFs may be diagnosed early using elevated MCV and reticulocyte counts, a diagnostic approach that may lead to earlier intervention and improved prognosis.

## Key Messages

- Isolated thrombocytopenia carrying bone marrow failure-related germline variants (ITGV-BMFs) showed poorer response and prognosis.
- The median progress time of ITGV-BMFs was 7.25 years, with an incidence of 57.69% (15/26).
- ITGV-BMFs can be identified with elevated mean corpuscular volume (MCV) and reticulocyte (RET) in early stage.

## Introduction

Thrombocytopenia encompasses a spectrum of platelet disorders with various underlying causes. Isolated thrombocytopenia refers to a reduced platelet count without any irregularities in other blood cell types and without symptoms or signs of a systemic disorder [1]. Patients with inherited thrombocytopenia (IT), inherited bone marrow failure syndromes (IBMFS), myelodysplastic syndrome (MDS), or certain primary immune deficiencies may primarily present with thrombocytopenia [2,3]. Immune thrombocytopenia (ITP) is often considered once known causes have been ruled out [4]. In some instances, effective therapeutic interventions and observation of increased or normal megakaryocytes can aid in the diagnosis of ITP, a disease with an incidence of approximately 2–10 per 100,000 adults each year and a prevalence of 9–20 per 100,000 adults [2,5,6]. Currently, first-line treatment with steroids and intravenous immunoglobulin typically yields response rates ranging from 59% to 79% [7–9]. Nevertheless, a significant subset of patients continues to exhibit poor responses, with platelet counts consistently below 30,000/µL or less than twice their baseline levels [10]. In thrombocytopenia, genetic factors extend beyond IT-related processes, such as MKL1, PADI2, TTF2, and TUBB1 [11,12]. It is imperative to consider additional underlying factors or genetic variation. Immune-related germline variants may lead to an early but transient response to steroid treatment [13]. Previously, we reported and reviewed the incidence of FANC heterozygous germline variants in acquired aplastic anemia (AA), which may result in diverse responses to conventional treatments [14]. Recent studies indicate that germline variants, particularly in heterozygous states, significantly contribute to the pathogenesis and progression of acquired AA [15]. However, their role in patients presenting with isolated thrombocytopenia, which initially mimics classic ITP, remains poorly characterized. These patients isolated thrombocytopenia carrying bone marrow failure-related germline variants (ITGV-BMFs) and are at risk of progressing to AA or MDS, posing significant diagnostic and therapeutic challenges. Early identification of these individuals through routine clinical indicators is therefore critical to prevent delayed management. Whole exome sequencing (WES) and next-generation sequencing are pivotal in uncovering underlying genetic causes. However, the substantial cost of sequencing has constituted a financial barrier for some patients. Our study aimed to compare patients with ITGV-BMFs to those with classic ITP. The objective was to identify sensitive and effective indicators to facilitate a faster and more affordable diagnostic process.

## Method

### Study design

Data from patients with ITGV-BMFs at the First Affiliated Hospital of Zhejiang Chinese Medical University between August 2018 and February 2024 were retrospectively reviewed. Patients diagnosed with classic ITP during the same period at our hospital were included as the control group. Diagnosis and disease assessment were based on the Chinese guidelines for the treatment of adult primary immune thrombocytopenia [16]. Informed consent was obtained in accordance with the Helsinki Declaration.

### Ethics approval and consent to participate

This study was approved by the ethical committee of the First Affiliated Hospital of Zhejiang Chinese Medical University (2024-KLS-325-02), and has been registered at *chictr.org.cn under* # ChiCTR2400085477. All procedures performed in studies involving human participants were in accordance with the ethical standards of the institutional and/or national research committee and with the 1964 Helsinki Declaration and its later amendments or comparable ethical standards. All patients had previously signed a hospital-wide general consent form during their initial visit, permitting the use of their anonymized data for future medical research. Written informed consent was obtained from all the participants.

### Patient selection

*Inclusion criteria of classic ITP* were (1) Diagnosis according to the Chinese guidelines for the treatment of adult primary immune thrombocytopenia [16]; (2) Patients who responded effectively to first-line ITP treatment; (3) Absence of disease progression or transformation during the follow-up period.

*Inclusion criteria of ITGV-BMFs* were (1) Patients with a prior diagnosis of ITP [17] and confirmed thrombocytopenia; (2) These patients underwent WES test; (3) Patients who did not achieve complete response (CR) after treatment or were under observation due to not meeting the criteria for initiating treatment.

*Exclusion criteria* were: (1) Patients with known prior causes of thrombocytopenia (e.g. inherited thrombocytopenia, secondary thrombocytopenia due to MDS, hypersplenism, drugs/infections/vaccine-induced thrombocytopenia, Evans Syndrome, active bleeding, and concomitant nutritional deficiencies, etc.); (2) Patients with other diseases that could affect the results (e.g. autoimmune diseases, malignancies, etc.); (3) Patients with other confirmed inherited diseases; (4) Patients with incomplete clinical or follow-up data.

### Baseline characteristics of patients

The baseline characteristics of patients at the initial diagnosis of ITP were systematically documented, including age, age at onset, gender, bleeding score (according to the Chinese guideline on the diagnosis and management of adult primary immune thrombocytopenia (version 2020)) [16], routine blood tests, reticulocyte (RET), and CD34% in bone marrow (BM) samples were reviewed and recorded.

### WES data generation and analysis

Bone marrow or peripheral blood samples were collected from ITGV-BMFs patients, and WES was conducted to assess the status of germline variants [18]. The analysis concentrated on 189 genes associated with IBMFs and IT [11,14,19,20]. Comprehensive details of the specific genes and the detailed clinical data of patients with gene mutations can be found in the supplementary materials (Supplementary Tables 1 and 2, respectively). The identified variants were classified according to their functional descriptions in the UniProt database [https://www.uniprot.org/uniprot/]. All detected variants were further confirmed as heterozygous germline mutations through rechecking oral mucous samples using PCR sequencing-based typing (PCR-SBT).

**Table 1.** Comparison of baseline characteristics at the initial diagnosis of ITP and prior therapies.

	ITGV-BMFs (*n* = 26)	Classic ITP (*n* = 33)	*p*
Age, years	27 (6–71)	52 (13–75)	< 0.001***
Age at onset, years	16.5 (4–61)	47 (12–75)	< 0.001***
Gender/male, *n* (%)	12 (46.15%)	12 (41.4%)	0.229
Bleeding score	1 (0–7)	4 (1–7)	< 0.001***
WBC, ×10^9^/L	5.0 (3.7–9.5)	4.7 (3.7–8.2)	0.829
Hb, ×g/L	126.5 (85–176)	129 (98–156)	0.894
PLT, ×10^9^/L	31 (12–95)	11 (2–44)	0.119
PCT, %	0.03 (0.01–0.11)	0.0355 (0.003–0.06)	0.638
MPV, fL	9.3 (7.1–17.8)	10.5 (8.4–13.1)	0.117
PDW, %	17.5 (7.7–19.7)	17.3 (13.3–21.6)	0.613
HCT, %	36.95 (25.1–54.6)	38.9 (28.6–47.0)	0.638
MCV, fL	101.3 (81.3–111.8)	87.3 (70.8–98.4)	< 0.001***
MCH, pg	34.6 (27.5–38.2)	28.7 (23.1–34.2)	< 0.001***
MCHC, g/L	338 (322–354)	332 (311–360)	0.34
RDW, %	13.5 (12.1–18.5)	13.3 (10.8–21.8)	0.613
RET, %	1.8 (0.47–3.48)	1.36 (0.5–2.73)	0.047*
CD34, %	0.15 (0.01–2.69)	0.93 (0.52–2.62)	0.001*
Prior therapy, *n* (%)			
GCs	17 (65.38%)	29 (87.88%)	–
IVIg	5 (19.23%)	6 (18.18%)	–
TPO-RA	11 (42.31%)	10 (30.30%)	–
rhTPO	4 (15.38%)	8 (24.24%)	–
CsA	13 (50.00%)	4 (12.12%)	–
TU	4 (15.38%)	0 (0%)	–
Danazol	4 (15.38%)	0 (0%)	–
Stanozol	1 (3.85%)	0 (0%)	–
Azathioprine	1 (3.85%)	0 (0%)	–
CHM	12 (46.15%)	10 (30.30%)	–

*Note*. ITGV-BMFs, isolated thrombocytopenia carrying bone marrow failure-related germline variants; ITP, immune thrombocytopenia; WBC, white blood cell; Hb, hemoglobin; PLT, platelet count; PCT, platelet crit; MPV, mean platelet volume; PDW, platelet distribution width; HCT, hematocrit; MCV, mean corpuscular volume; MCH, mean corpuscular hemoglobin; MCHC, mean corpuscular hemoglobin concentration; RDW, red blood cell distribution width; RET, reticulocytes; GCs, Glucocorticoids; IVIg, intravenous immunoglobulin; TPO-RA, thrombopoietin receptor agonist. rhTPO, recombinant human thrombopoietin; CsA, cyclosporine; TU, testosterone undecanoate; CHM, Chinese herbal medicine; *<0.05, ***<0.001.

**Table 2.** Analysis of clinical available indexes for ITGV-BMFs patients using logistic regression.

Characteristics	Univariate		Multivariate	
	HR and 95%CI	*p*	HR and 95%CI	*p*
Age at onset	0.881 (0.831–0.935)	<0.001***	0.851 (0.771–0.972)	0.014*
Bleeding score	0.506 (0.330–0.774)	0.002*	0.187 (0.034–0.982)	0.048*
PCT	7.589 (0.000–15.715)	0.595	–	–
MPV	0.743 (0.536–1.053)	0.094	–	–
PDW	0.928 (0.709–1.248)	0.608	–	–
HCT	0.933 (0.863–1.014)	0.093	–	–
MCV	1.184 (1.085–1.292)	<0.001***	1.369 (0.649–2.887)	0.409
MCH	1.389 (1.144–1.686)	0.001*	0.639 (0.081–5.022)	0.671
MCHC	1.036 (0.970–1.110)	0.276	–	–
RDW	0.882 (0.635–1.225)	0.454	–	–
RET	3.155 (1.009–9.861)	0.048*	1.372 (0.038–49.133)	0.863
CD34	0.017 (0.001–0.288)	0.005*	1.393 (0.012–167.098)	0.892

*Note*. ITGV-BMFs, isolated thrombocytopenia carrying bone marrow failure-related germline variants; PCT, platelet crit; MPV, mean platelet volume; PDW, platelet distribution width; HCT, hematocrit; MCV, mean corpuscular volume; MCH, mean corpuscular hemoglobin; MCHC, mean corpuscular hemoglobin concentration; RDW, red blood cell distribution width; RET, reticulocytes; ***p < 0.001, *p < 0.05.

### ACMG classification

The assessment of the potential pathogenicity of the variants was conducted using the American College of Medical Genetics and Genomics (ACMG) classification system and the ClinVar database (https://www.ncbi.nlm.nih.gov/clinvar/). Variants were classified as pathogenic, likely pathogenic, uncertain significance, likely benign, or benign [21].

### Follow-up and outcome assessment

Patient follow-up duration extended from the date of initial ITP diagnosis to August 2024. During data collection, patients with missing information were identified, and their contact details were retrieved from medical records. Follow-up telephone calls were then conducted to obtain the missing data. The primary endpoint was progression-free survival (PFS), which is defined as the time from the ITP diagnosis to the development of AA or MDS. The treatment response was defined as follows[16]: complete response (CR): Platelet count (PLT) ≥100 × 10^9^/L, with no bleeding manifestations; response (R): PLT ≥30 × 10^9^/L and at least doubling of the baseline count, with no bleeding manifestations; No response (NR): PLT <30 × 10^9^/L, or not achieving at least doubling of the baseline count, or with bleeding manifestations. Our follow-up for all patients was greater than or equal to 6 months.

### Statistical analysis

Data analysis was conducted using IBM SPSS version 23.0 and GraphPad Prism version 8.3.0 software were utilized for data analysis. The Shapiro–Wilk test was employed to evaluate the normality of quantitative data. Parametric tests were employed for quantitative data with normal distribution, while non-parametric tests were utilized for data with non-normal distribution. The Chi-square test was applied for categorical variables with more than two levels, and binary logistic regression was used to analyze influencing factors. One-way analysis of variance and the least significant difference (LSD) method were employed for comparisons of means among multiple groups. Quantitative data following a normal distribution were expressed as mean ± standard deviation (SD), while data with a skewed distribution were represented as median (range). A significance level of *p* < 0.05 was considered statistically significant. Receiver-operating characteristic (ROC) curves were constructed to assess the discriminatory performance among different groups of thrombocytopenia, with the area under the ROC curve (AUC) used as an evaluation metric.

## Results

### Patient characteristics

**Figure 1.**
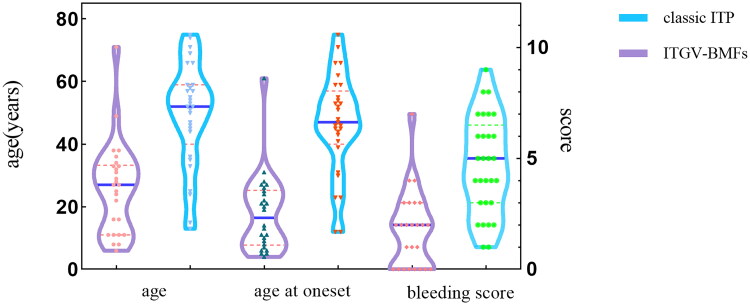
The age, onset age, and bleeding score in patients with ITGV-BMFs and classic ITP at baseline. The violin plot demonstrates the median and distribution. The central blue horizontal line in each violin plot represents the median, and the pink dotted lines indicate the first and third quartiles, respectively. The symbol depicts the distribution of individual values.

**Figure 2.**
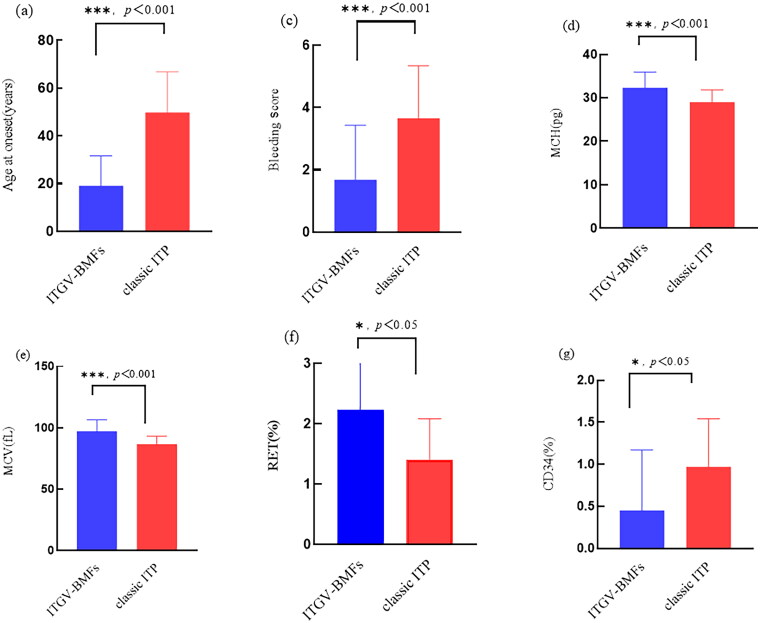
Comparison of general conditions and blood routine in the patients with ITGV-BMFs and classic ITP. Panels (a)–(g) show statistically significant indicators. Note: (a) Age at onset; (b) duration of illness; (c) bleeding score; (d) MCH; (e) MCV; (f) RET; (g) CD34. *<0.05, ***<0.001.

A cohort of 59 isolated thrombocytopenia patients were included in this study. Of these, 26 patients underwent WES analysis for ITGV-BMFs, while 33 patients constituted the control group, with a confirmed diagnosis of ITP and a median follow-up duration of 7.25 years. Baseline characteristics of the two groups were compared (Table 1). The median age was 27 years for the ITGV-BMFs group and 52 years for the classic ITP group, with median ages at onset being 16.5 years and 47 years, respectively, both differences were statistically significant (*p* < 0.001). The baseline bleeding score of ITGV-BMFs patients was lower than that of classic ITP (*p* < 0.001) (Table 1; Figure 1). Furthermore, a comparative analysis of pre-treatment laboratory indicators revealed that ITGV-BMFs patients exhibited lower CD34% in bone marrow, higher MCV, MCH, and RET than those with classic ITP (*p* < 0.001) (Table 1; Figure 2).

### Distribution of germline variants in patients with ITGV-BMFs

A total of 37 germline variants were observed in our cohort. The most frequently observed germline variant was *FANCA* accounting for 10.81% (4/37), followed by *SLX4* 8.11% (3/37), following with *ERCC4, BLM, TERT, PALB2, FANCD2*, and *BRCA2* (5.41%, 2/37) (Supplementary Figure 1a). Functional annotation of these genes revealed their involvement in DNA damage and repair (*n* = 27, 72.97%), telomere biology (*n* = 3, 8.11%), based on data from the UniProt database (Supplementary Figure 1b). Additionally, 9 out of 26 patients with ITGV-BMFs were found to have a combination of somatic mutations, including *BCOR*, *CEBPA*, *TET2*, and others.

### Clinical available indexes may contribute to predicting ITGV-BMFs diagnosis

The contribution of each variable to the risk assessment of germline variants was analyzed using univariate analysis, with results expressed as odds ratios (OR). Factors such as age at onset, bleeding score, MCV, MCH, RET, CD34%, and response to treatment were identified as predictors for distinguishing patients with ITGV-BMFs (*p* < 0.05). In the final multivariate logistic regression analysis indicated that the patients with ITGV-BMFs may exhibit a specificity characteristic with earlier onset age (OR = 0.851, 95% confidence interval (CI) 0.771–0.972, *p* = 0.014) and lower bleeding score (OR = 0.187, 95% CI 0.034–0.982, *p* = 0.048) (Table 2).

**Figure 3.**
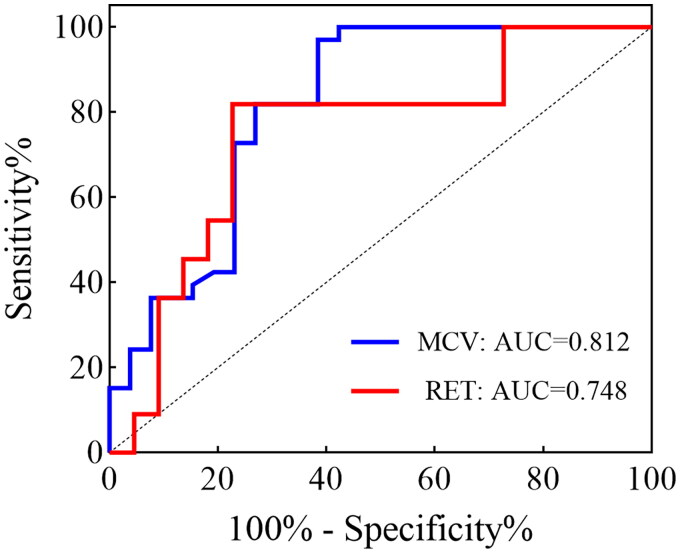
ROC curve illustrating the diagnostic value of MCV (a) and RET (b) in patients with ITGV-BMFs. ROC, receiver operating characteristic; AUC, area under the ROC curve. ROC curve with specificity on the *x* axis and sensitivity on the *y* axis.

We further assessed the correlation of age at onset, bleeding score, MCV, MCH, RET and CD34% with the occurrence of germline variants through ROC analysis. The results showed that the AUC for MCV was 0.812 (95% CI = 0.694–0.930, *p* < 0.0001), with an optimal cut-off value of 97.35 fL, as determined by maximizing the Youden index. This cut-off value corresponded to a sensitivity of 0.615 and a specificity of 0.97. respectively. The AUC value for RET was 0.748 (95% CI = 0.566–0.930, *p* = 0.02), and the optimal cut-off value for RET, determined by maximizing the Youden index, was 1.645%, with the corresponding sensitivity and specificity of 0.773 and 0.409, respectively (Figure 3). Except for MCV and RET, the AUC value of other indicators is less than 0.5 with no significant differences.

### Outcomes of ITGV-BMFs

**Figure 4.**
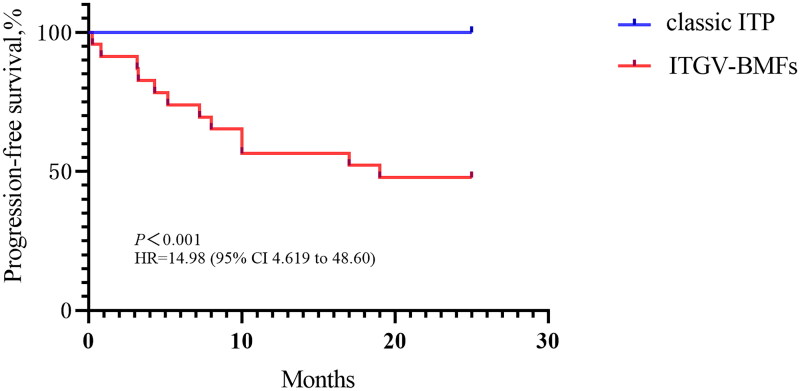
Kaplan–Meier estimates of follow-up time from initial diagnosis to disease transformation, including progression to AA or MDS. Hazard ratio, 14.98 (95% CI 4.619–48.60; *p* < 0.001).

The median follow-up period was 7.125 years for patients with ITGV-BMFs and 7.41 years for those with classic ITP. During the follow-up period, 13/26 (50.00%) patients were diagnosed with AA, and 2/26 (7.69%) with MDS in ITGV-BMFs patients, while no cases of AA or MDS were observed in the classic ITP group. Patients with ITGV-BMFs exhibited a median PFS of 7.25 years (hazard ratio [HR] = 14.98, *p* < 0.001) (Figure 4). In a cohort of patients with ITGV-BMFs undergoing multiple lines of treatment, 6 out of 26 individuals (23.08%) achieved CR. However, it is noteworthy that 3 of these patients subsequently experienced relapse. 3 out of 26 patients (11.54%) attained remission, with one experiencing a subsequent relapse. The majority, 17 out of 26 patients (65.38%), did not respond to any treatments.

## Discussion

ITP, an autoimmune disease, often manifests with hemorrhage as the primary clinical symptom [22]. Despite advancements in treatments, a small proportion of patients remain inadequately managed with these therapies [23,24]. It is hypothesized that genetic variants may play a significantly role in suboptimal therapeutic outcomes. WES has identified a heterozygous p.G76S mutation in the *TNFRSF13B* gene, which is considered a negative prognostic factor for ITP treatments [25]. However, the high costs and extended waiting periods remain challenging for patients. This study offers potential indicators for better identification of these patients, aiming to improve the efficiency of diagnosis and treatment.

Poor response in some ITP patients may be associated with their genomic predisposition. Our study identified a notable frequency (24.32%) of heterozygous germline mutations in the FANC gene family among patients with ITGV-BMFs at our center. This frequency is significantly lower than that observed in acquired AA (45.9%) [14], suggesting only a partial occurrence of disease transformation. Studies have shown that *FANC* variants increase susceptibility to develop AA/MDS [26], but further investigation is required to explore their implications in ITP. The progression of some patients indicates that thrombocytopenia may represent a prodromal stage of *FANC*-associated bone marrow failure syndromes. In this study, we found that the majority of germline variants identified in patients with ITGV-BMFs are associated with DNA damage and repair mechanisms. The FA (Fanconi anemia) pathway plays a crucial role in DNA repair. Although association between FA and ITP is rarely reported, existing evidence indicates suggests that dysregulation caused by *FANC* mutations may result in variable immunodeficiency or chromosomal alterations [27]. Such chromosomal aberrations may arise from the activation of alternative, error-prone DNA repair pathways [28]. Additionally, patients with ITGV-BMFs may have abnormalities in telomere biology, including critically short telomere and germline variants in gene such as *TERT* or *NOP10*, which are linked to inherited bone marrow failure syndromes [29]. Furthermore, in this study, somatic mutations were also found in some patients with ITGV-BMFs, potentially contributing to poor efficacy. A significant increase in the demand for second-line treatment among some ITP patients appears to be associated with somatic mutations such as *IFNA17*, *IFNLR1*, and *REL* [30]. Previous research has shown that ITP patients harboring mutations in genes such as *DNMT3A*, *TET2*, *ASXL1*, *BCOR*, *PIGA*, and *U2AF1* tend to progress into other clonal hematologic disorders, leading to treatment ineffectiveness [31]. Even though some germline variants are of uncertain significance (VUS), their enrichment remains worth exploring. As our previous studies suggested, VUS in genes like *FANC* can impact disease severity and treatment outcomes, which needs further investigation [14,15].

These individuals with ITGV-BMFs exhibited decreased bleeding scores and lower CD34 cell counts, as well as increased RET levels, which differ from the high bleeding scores and normal CD34 cell counts typically observed in classic ITP. In general, most patients with AA or ITP are within the normal range for MCV and MCH levels. In contrast, in patients with MDS, the MCH and MCV values are often slightly elevated [32]. Additionally, there have been reports describing the median MCV levels in the NSAA (non-severe aplastic anemia) and MDS groups being higher than those in the SAA (severe aplastic anemia) group [33]. However, to the best of our knowledge, the characteristic features of MCV and MCH in ITGV-BMFs patients have not been previously explored or reported. Our study is the first to report that individuals with ITGV-BMFs exhibit increased MCV and mean corpuscular hemoglobin (MCH) levels, even in the early stages prior to the onset of thrombocytopenia. Particularly, MCV and RET showed good predictive strength, as indicated by ROC analysis. A study suggests that within the NSAA cohort, 59.1% of patients exhibited macrocytic anemia, indicating that clinicians should not exclude AA when encountering elevated MCV [33]. Additionally, increased MCV is accepted as a prognostic factor for the diagnosis of hematological diseases, especially for MDS/AML[33,34]. Herein, patients with ITGV-BMFs may have a risk of developing acquired AA or MDS, as suggested by follow-up with 15 (57.69%) individuals re-diagnosed with AA/MDS due to poor treatment response. Early indicators such as decreased bone marrow proliferation and abnormal erythrocytes may already exist in these individuals. Utilizing the indicators we found (such as MCV, RET, MCH, etc.) may aid in the early recognition of these patients.

One limitation of this study is the restricted sample size, WES results for patients with classic ITP. In light of this limitation, we intend to expand the sample size of research participants to establish a predictive model that would contribute to personalized treatment strategies and improve patient outcomes. Additionally, we propose conducting WES examinations in future studies, contingent upon securing further funding.

## Conclusion

The presence of germline variant may be a significant factor for a portion of thrombocytopenia patients with poor response. Our study revealed various characteristics of ITGV-BMFs, such as age at onset, bleeding scores, MCV, CD34%, and RET, which could potentially be used as predictors to replace WES eventually. As such, ITGV-BMFs may be diagnosed early using elevated MCV and reticulocyte counts, leading to earlier interventions and improved prognosis.

## Supplementary Material

Supplementary Figure 1.tif

Supplementary_Table_2 - Clean.docx

Supplementary_Table_1 - Clean.docx

## Data Availability

The data used and/or analyzed during the current study are available from the corresponding author upon a reasonable request.

## Supplementary

**Supplementary Figure 1.** Frequencies and identified genes in the ITGV-BMFs cohort. (a) Frequencies and specific gene variants in the ITGV-BMFs cohort. (b) Gene functional annotation and classification.

## References

[1] Kosmidou A, Gavriilaki E, Tragiannidis A. The challenge for a correct diagnosis of refractory thrombocytopenia: ITP or MDS with isolated thrombocytopenia? Cancers (Basel). 2024;16(8):1462. doi: 10.3390/cancers16081462. 38672544 PMC11048195

[2] Miltiadous O, Hou M, Bussel JB. Identifying and treating refractory ITP: difficulty in diagnosis and role of combination treatment. Blood. 2020;135(7):472–490. doi: 10.1182/blood.2019003599. 31756253 PMC7484752

[3] Grace RF, Lambert MP. An update on pediatric ITP: differentiating primary ITP, IPD, and PID. Blood. 2022;140(6):542–555. doi: 10.1182/blood.2020006480. 34479363

[4] Sachs UJ. Diagnosing immune thrombocytopenia. Hamostaseologie. 2019;39(3):250–258. doi: 10.1055/s-0039-1678739. 30763966

[5] Lv Y, Shi H, Liu H, et al. Current therapeutic strategies and perspectives in refractory ITP: what have we learned recently? Front Immunol. 2022;13:953716. doi: 10.3389/fimmu.2022.953716. 36003388 PMC9393521

[6] Liu XG, Hou Y, Hou M. How we treat primary immune thrombocytopenia in adults. J Hematol Oncol. 2023;16(1):4. doi: 10.1186/s13045-023-01401-z. 36658588 PMC9850343

[7] Wei Y, Ji XB, Wang YW, et al. High-dose dexamethasone vs prednisone for treatment of adult immune thrombocytopenia: a prospective multicenter randomized trial. Blood. 2016;127(3):296–302; quiz 370. doi: 10.1182/blood-2015-07-659656. 26480931

[8] Mithoowani S, Arnold DM. First-line therapy for immune thrombocytopenia. Hamostaseologie. 2019;39(3):259–265. doi: 10.1055/s-0039-1684031. 31170773

[9] Kado R, McCune WJ. Treatment of primary and secondary immune thrombocytopenia. Curr Opin Rheumatol. 2019;31(3):213–222. doi: 10.1097/BOR.0000000000000599. 30920453

[10] Rodeghiero F, Stasi R, Gernsheimer T, et al. Standardization of terminology, definitions and outcome criteria in immune thrombocytopenic purpura of adults and children: report from an international working group. Blood. 2009;113(11):2386–2393. doi: 10.1182/blood-2008-07-162503. 19005182

[11] Johnson B, Lowe GC, Futterer J, et al. Whole exome sequencing identifies genetic variants in inherited thrombocytopenia with secondary qualitative function defects. Haematologica. 2016;101(10):1170–1179. doi: 10.3324/haematol.2016.146316. 27479822 PMC5046646

[12] Mekchay P, Ittiwut C, Ittiwut R, et al. Whole exome sequencing for diagnosis of hereditary thrombocytopenia. Medicine (Baltimore). 2020;99(47):e23275. doi: 10.1097/MD.0000000000023275. 33217855 PMC7676547

[13] Liu Y, Gusev A, Heng YJ, et al. Somatic mutational profiles and germline polygenic risk scores in human cancer. Genome Med. 2022;14(1):14. doi: 10.1186/s13073-022-01016-y. 35144655 PMC8832866

[14] Shen Y, Liu Q, Li H, et al. Whole-exome sequencing identifies FANC heterozygous germline mutation as an adverse factor for immunosuppressive therapy in Chinese aplastic anemia patients aged 40 or younger: a single-center retrospective study. Ann Hematol. 2023;102(3):503–517. doi: 10.1007/s00277-023-05086-9. 36622392 PMC9977704

[15] Wang P, Jiang W, Lai T, et al. Germline variants in acquired aplastic anemia: current knowledge and future perspectives. Haematologica. 2024;109(9):2778–2789. doi: 10.3324/haematol.2023.284312. 38988263 PMC11367197

[16] Thrombosis C, Hemostasis Group. [Chinese guideline on the diagnosis and management of adult primary immune thrombocytopenia (version 2020)]. Zhonghua Xue Ye Xue Za Zhi. 2020;41(8):617–623. 32942813 10.3760/cma.j.issn.0253-2727.2020.08.001 PMC7525165

[17] Provan D, Arnold DM, Bussel JB, et al. Updated international consensus report on the investigation and management of primary immune thrombocytopenia. Blood Adv. 2019;3(22):3780–3817. doi: 10.1182/bloodadvances.2019000812. 31770441 PMC6880896

[18] Nalepa G, Clapp DW. Fanconi anaemia and cancer: an intricate relationship. Nat Rev Cancer. 2018;18(3):168–185. doi: 10.1038/nrc.2017.116. 29376519

[19] McReynolds LJ, Rafati M, Wang Y, et al. Genetic testing in severe aplastic anemia is required for optimal hematopoietic cell transplant outcomes. Blood. 2022;140(8):909–921. doi: 10.1182/blood.2022016508. 35776903 PMC9412004

[20] Trotta L, Norberg A, Taskinen M, et al. Diagnostics of rare disorders: whole-exome sequencing deciphering locus heterogeneity in telomere biology disorders. Orphanet J Rare Dis. 2018;13(1):139. doi: 10.1186/s13023-018-0864-9. 30115091 PMC6097299

[21] Richards S, Aziz N, Bale S, et al. A.L.Q.A. Committee, Standards and guidelines for the interpretation of sequence variants: a joint consensus recommendation of the American College of Medical Genetics and Genomics and the Association for Molecular Pathology. Genet Med. 2015;17(5):405–424. doi: 10.1038/gim.2015.30. 25741868 PMC4544753

[22] Zhan Y, Cao J, Ji L, et al. Impaired mitochondria of Tregs decreases OXPHOS-derived ATP in primary immune thrombocytopenia with positive plasma pathogens detected by metagenomic sequencing. Exp Hematol Oncol. 2022;11(1):48. doi: 10.1186/s40164-022-00304-y. 36050760 PMC9434515

[23] Al-Samkari H, Neufeld EJ. Novel therapeutics and future directions for refractory immune thrombocytopenia. Br J Haematol. 2023;203(1):65–78. doi: 10.1111/bjh.19078. 37735554 PMC11101754

[24] Schifferli A, Le Gavrian G, Aladjidi N, et al. Chronic refractory immune thrombocytopenia in adolescents and young adults. Br J Haematol. 2023;203(1):36–42. doi: 10.1111/bjh.19081. 37735549

[25] Peng HL, Zhang Y, Sun NN, et al. A gain-of-function mutation in TNFRSF13B is a candidate for predisposition to familial or sporadic immune thrombocytopenia. J Thromb Haemost. 2017;15(11):2259–2269. doi: 10.1111/jth.13806. 28834165

[26] Sekinaka Y, Mitsuiki N, Imai K, et al. Common variable immunodeficiency caused by FANC mutations. J Clin Immunol. 2017;37(5):434–444. doi: 10.1007/s10875-017-0396-4. 28493158

[27] García-de-Teresa B, Rodríguez A, Frias S. Chromosome instability in Fanconi anemia: from breaks to phenotypic consequences. Genes (Basel). 2020;11(12):1528. doi: 10.3390/genes11121528. 33371494 PMC7767525

[28] Natarajan AT, Palitti F. DNA repair and chromosomal alterations. Mutat Res. 2008;657(1):3–7. doi: 10.1016/j.mrgentox.2008.08.017. 18801460

[29] Savage SA, Giri N, Baerlocher GM, et al. Alter, TINF2, a component of the Shelterin telomere protection complex, is mutated in dyskeratosis congenita. Am J Hum Genet. 2008;82(2):501–509. doi: 10.1016/j.ajhg.2007.10.004. 18252230 PMC2427222

[30] Despotovic PLM, Flanagan JM, Bennett JM, et al. Genes influencing the development and severity of chronic ITP identified through whole exome sequencing. Blood. 2015;126(23):73–73. doi: 10.1182/blood.V126.23.73.73.

[31] Wang Y, Yu T, Dong Q, et al. Clonal hematopoiesis in primary immune thrombocytopenia. Blood Cancer J. 2022;12(3):40. doi: 10.1038/s41408-022-00641-5. 35293382 PMC8924250

[32] Cascio MJ, DeLoughery TG. Anemia: evaluation and diagnostic tests. Med Clin North Am. 2017;101(2):263–284. doi: 10.1016/j.mcna.2016.09.003. 28189170

[33] Liu L, Fu Q, Zhang D, et al. Analysis of mean corpuscular volume and red cell distribution width in patients with aplastic anemia. Hemoglobin. 2023;47(2):31–35. doi: 10.1080/03630269.2023.2206575. 37161838

[34] Takahashi N, Kameoka J, Takahashi N, et al. Causes of macrocytic anemia among 628 patients: mean corpuscular volumes of 114 and 130 fL as critical markers for categorization. Int J Hematol. 2016;104(3):344–357. doi: 10.1007/s12185-016-2043-x. 27352093

