# Supplementary figures and images for: Clinical characteristic of isolated thrombocytopenia in patients with bone marrow failure-related germline variants: a retrospective study from a single centre
Source: Ann Med. 2025 Jun 26;57(1):2523560. doi: 10.1080/07853890.2025.2523560 (PMC12203705; doi:10.1080/07853890.2025.2523560)

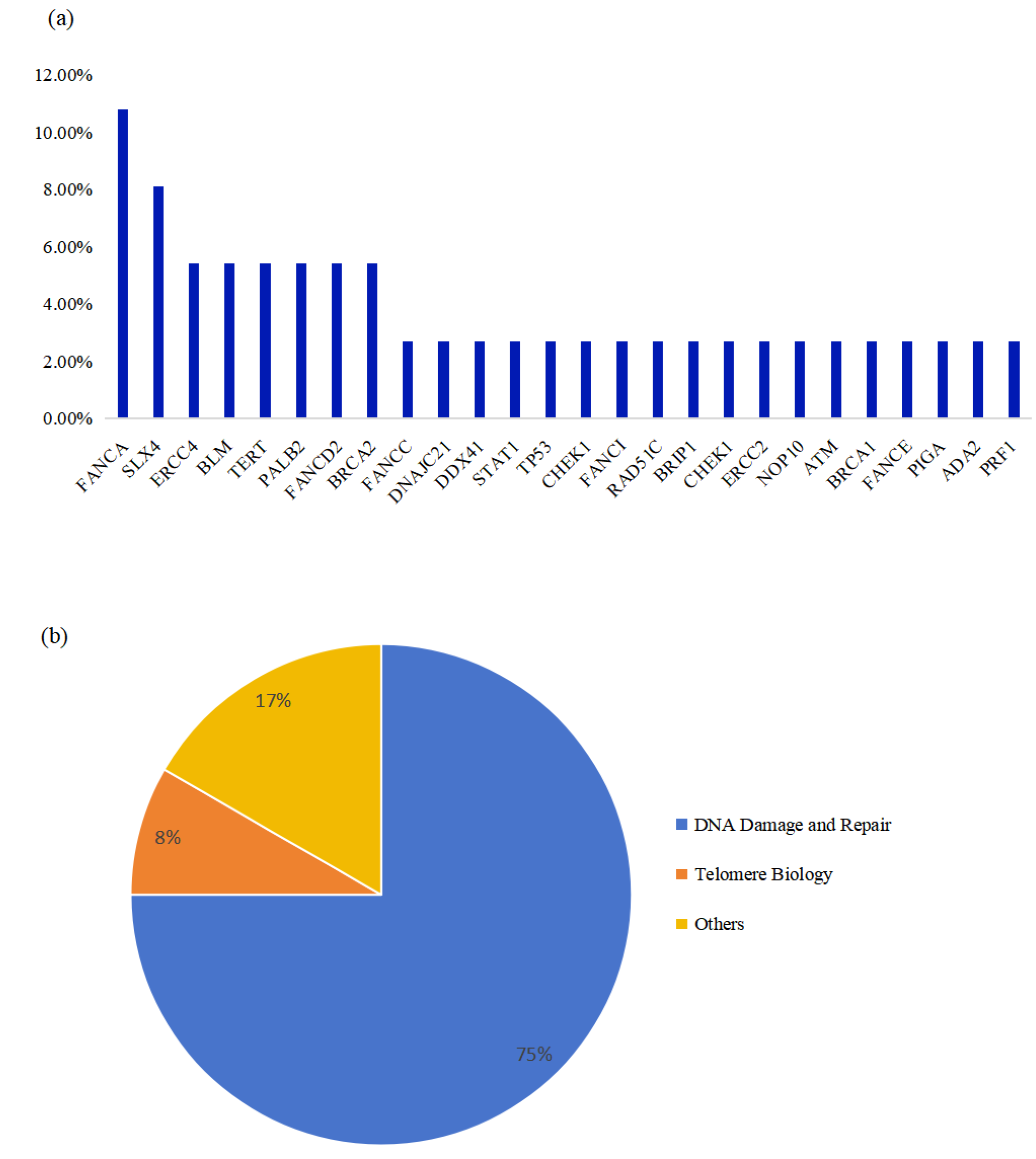

Supplement: Supplementary Figure 1.tif [file IANN_A_2523560_SM8664.tif]
