# Supplementary material for: Clinical characteristic of isolated thrombocytopenia in patients with bone marrow failure-related germline variants: a retrospective study from a single centre
Source: Ann Med. 2025 Jun 26;57(1):2523560. doi: 10.1080/07853890.2025.2523560 (PMC12203705; doi:10.1080/07853890.2025.2523560)
Supplement: Supplementary_Table_2 - Clean.docx [file IANN_A_2523560_SM8663.docx]

**Supplementary Table 2. Detailed clinical data and ACMG classification of patients with ITGV-BMFs**

| Patient ID | Age diagnosed with thrombocytopenia, years | Germline Variants type,VAF%  / Clinical interpretation | Germline VAF (%)/ Clinical interpretation | Frequency in the population group | Somatic mutation type | Somatic VAF (%) | List of previous therapies | Effective Medication | outcome | Updated diagnosis (AA=1,MDS=2) | Follow-up time from initial diagnosis to disease transformation, years |
| --- | --- | --- | --- | --- | --- | --- | --- | --- | --- | --- | --- |
| P01 | 16 | DNAJC21,exon4,c.1879C>T,p.R627C | 30%  Pathogenic | DNAJC21:0.002(KG) | - | - | GCs, Danazol, CHM | - | NR | 1 | 6.8 |
| P02 | 38 | DDX41,exon8,c.656G>C,p.R219P | 46.47%  Pathogenic | DDX41:- | - | - | Watch and wait, Danazol, CHM, TPO-RA | - | NR | 2 | 6 |
| P03 | 71 | TP53,exon4,c.215C>G,p.P72R | 47.44%  Pathogenic | TP53:- | TET2, TP53, SH2B3 | Unavailable | GCs, rhTPO | GCs + rhTPO | CR | 0 | - |
| P04 | 29 | FANCI,exon12,c.1111A>G,p.S371G RAD51C,exon6,c.862A>G,p.T288A | 48.89%  Conflicting  51.4%  Uncertain significance | FANCI:0.000199681(KG)  RAD51C:- | - | - | GCs, CHM, CsA | - | NR | 0 | - |
| P05 | 27 | STAT1,exon22,c.1957G>A,p.V653I | 2.86%  Uncertain significance | STAT1:- | BCOR,  CEBPA, ARID1B,RUNX1 | 14.2%  39.3%  46.2%  47.9% | Watch and wait, GCs, CsA | - | NR | 1 | 10 |
| P06 | 33 | ERCC4,exon3,c.389-1G>A FANCE,exon2,c.C743A,p.A248E SLX4,exon7,c.G1442A,p.R481Q | 50.89%  Uncertain significance  43.65%  Uncertain significance  51.47%  Uncertain significance | ERCC4:-  FANCE:-  SLX4:0.0002(KG) | - | - | GCs, TPO-RA, TU | - | NR | 1 | 8 |
| P07 | 24 | SLX4,exon12,c.2776T>C,p.C926R | 45.80%  Uncertain significance | SLX4:0.000199681(KG) | BCOR | 24.62% | GCs + TPO-RA, CsA, Azathioprine | - | NR | 0 | - |
| P08 | 8 | BLM,exon12,c.2515A>G,p.K839E BRIP1,exon8,c.1018C>T,p.L340F CHEK1,exon1,c.99G>C,p.W33C ERCC2,exon8,c.691G>A,p.V231M | 54.40%  Pathogenic  46.20%  Uncertain significance  41.70%  Pathogenic  44.4%  Pathogenic | BLM:0.000173(ExAC)  BRIP1:0.000016(ExAC)  CHEK1:-  ERCC2:0.000126(ExAC), 0.000128(gnomAD) | ABL1, BLM, KMT2C | 49.70%  49.10%  4.10% | IVIG, GCs, TPO-RA | GCs + TPO-RA | CR, relapse | 0 | - |
| P09 | 32 | ATM,exon57,c.8377C>A,p.P2793T | 48.10%  Pathogenic | ATM:- | BCOR, CEBPA, RB1, TNFAIP3 | 26.00%  60.50%  48.10%  49.50% | GCs, CsA, TPO-RA, CHM | TPO-RA | R | 1 | 10 |
| P10 | 27 | FANCA,exon40,c.3973G>A,p.D1325N | 47.40%  Uncertain significance | FANCA:- | KMT2C | 41.00% | GCs, TU, CHM | - | NR | 0 | - |
| P11 | 33 | FANCC,exon11,c.1000C>T,p.R334W SLX4,exon15,c.5248_5249delinsTT,p.A1750L | 48.20%  Pathogenic  51.50%  Uncertain significance | FANCC:0.000118(ExAC)  SLX4:- | BCOR | 1.87% | Watch and wait, danazol, CsA | - | NR | 1 | 3.17 |
| P12 | 25 | BRCA2,exon11,c.6325G>A,p.V2109I | 55.2%  likely benign | BRCA2:0.000275(ExAC),0.000233(gnomAD) | TET2, CREBBP | 49.40%,  49.30% | CHM | - | NR | 1 | 0.25 |
| P13 | 34 | BLM,exon10,c.2293G>A p.V765I | 50.00%  Uncertain significance | BLM:0.000342(ExAC),0.000351(gnomAD) | BCOR, CEBPA, CUX1, FBXW7 | 60.39%  49.28%  46.82%  49.3 | CHM, Stanozol | - | NR | 0 | - |
| P14 | 11 | TERT,exon,c.1253C>T p.A418V | 48.40%  Uncertain significance | TERT:0.000014(gnomAD) | - | - | GCs, TPO-RA, CHM | GCs + TPO-RA | CR | 0 | - |
| P15 | 16 | TERT,exon4,c.1782G>C,p.K594N | 49.70%  Uncertain significance | TERT:- | - | - | IVIG, GCs, rhTPO, CsA, CHM | CsA + rhTPO | CR, relapse | 0 | - |
| P16 | 11 | ERCC4,exon8,c.1787C>A,p.A596E PALB2,exon8,c.1787C>A,p.A596E | 44.7%,  Uncertain significance  52.5%  Uncertain significance | ERCC4:-  PALB2:- | - | - | GCs, rhTPO, CsA | - | NR | 1 | 0.25 |
| P17 | 36 | FANCD2,exon25,c.A2372G,p.N791S | 43.64%  Uncertain significance | FANCD2:0.00007626(ExAC) | - | - | Watch and wait, CsA, TU | - | NR | 1 | 17 |
| P18 | 11 | PALB2,exon5,c.G2509A,p.E837K NOP10,exon2,c.T104A,p.F35Y | 56.25% Uncertain significance  57.28%  Uncertain significance | PALB2:0.0001(ExAC)  NOP10:- | - | - | Watch and wait, IVIG, GCs, CHM | IVIG + GCs | R | 1 | 5.17 |
| P19 | 22 | ATM,exon61,c.G8805A,p.M2935I | 41.58%  Uncertain significance | ATM:0.000025(ExAC),0.000036(gnomAD) | - | - | GCs, TPO-RA, rhTPO, CsA | - | NR | 1 | 3.25 |
| P20 | 38 | FNACA,exon23,c.2080G>A,p.D694N BRCA2,exon9,c.695A>G,p.Y232C | 46.70%  Uncertain significance55.30%  Uncertain significance | FNACA:0.000050(gnomAD)  BRCA2:0.000050(gnomAD) | BCORL1 | 49.50% | Watch and wait, CsA,TPO-RA | - | NR | 1 | 7.25 |
| P21 | 31 | FNACA,exon30,c.A2944G,p.T982A | 48.46%  Uncertain significance | FNACA:0.000016(ExAC),0.000020(gnomAD) | - | - | Watch and wait,  TU | - | NR | 1 | 4.33 |
| P22 | 8 | FANCD2,exon25,c.2372A>G,p.Asn262Lys | Het  *Uncertain significance* | FANCD2:0.000076(ExAC),0.000029(gnomAD) | - | - | CHM | - | NR | 0 | - |
| P23 | 52 | BRCA1,exon10,c.2726A>T,p.Asn909Ile SAMD9,exon3,c.848G>A,p.C283Y  SLX4,exon2,c.420G>T,p.E140D | 50.07%，  *Uncertain significance*  51.61%  Pathogenic  48.2%  Likely pathogenic | BRCA1  :0.000049(ExAC),0.000080(gnomAD)  SAMD9:-  SLX4:- | - | - | Watch and wait,  CsA, danazol | - | NR | 1 | 19 |
| P24 | 28 | PIGA,exon2,c.152G>A,p.(Ser51Asn)  ADA2,exon3,c.505C>T,p.(Arg169Trp) | 45.17%  *Uncertain significance*  51.50%  Likely pathogenic | PIGA:-  ADA2:0.000008(ExAC),0.000004(gnomAD) | - | - | GCs, IVIG, TPO-RA, CsA | GCs + TPO-RA | CR, relapse | 0 | - |
| P25 | 49 | FANCA,exon42,c.4225C>T:p. | 49.87%  Uncertain significance | FANCA:0.00019  9681(KG),0.0002(ExAC) | - | - | GCs, TPO-RA |  | R, relapse | 2 | 20 |
| P26 | 6 | PRF1,exon3,c.1620A>Gp.Gln540= | Het  Uncertain significance | PRF1:C=0.001890(ExAC),0.001913(gnomAD) | - | - | IVIG, GCs, CsA, TPO-RA，CHM | CsA + TPO-RA | CR | 0 | - |

Note: ACMG, American College of Medical Genetics and Genomics; ITGV-BMFs, isolated thrombocytopenia carrying bone marrow failure-related germline variants; VAF, variant allele frequencies Het, Heterozygous; GCs, Glucocorticoids; IVIG, intravenous immunoglobulin; rhTPO, recombination human thrombopoietin; CsA, cyclosporine; TPO-RA, thrompoietin-receptor agonists; TU, testosterone undecanoate; CHM, Chinese herbal medicine. Complete response (CR): Platelet count ≥100 × 109/L, with no bleeding manifestations; Response (R): Platelet count ≥30 × 10^9^/L and at least doubling of the baseline count, with no bleeding manifestations; No response (NR): Platelet count ＜30 × 10^9^/L, or not achieving at least doubling of the baseline count, or with bleeding manifestations. "Het" indicates heterozygous mutations reported by external laboratories, though detailed VAF were unavailable. Heterozygous variants with VAF between 15%-85% were confirmed by Sanger sequencing of germline samples (buccal swab or nail). "Unavailable" denotes somatic mutations without reported frequencies.
