# Supplementary material for: Clinical characteristic of isolated thrombocytopenia in patients with bone marrow failure-related germline variants: a retrospective study from a single centre
Source: Ann Med. 2025 Jun 26;57(1):2523560. doi: 10.1080/07853890.2025.2523560 (PMC12203705; doi:10.1080/07853890.2025.2523560)
Supplement: Supplementary_Table_1 - Clean.docx [file IANN_A_2523560_SM8662.docx]

**Supplementary Table 1. Significant genes of germline mutation in inherited bone marrow failure syndrome**

| Disease | Genes list of germline mutation |
| --- | --- |
| FA | FANCA, FANCC, FANCG, BRCA1, FANCD2, FANCF, FANCL, FANCI, SLX4, PALB2, FANCM, FANCE, FANCB, BRIP1, BRCA2, RAD51, ERCC4, UBE2T, RAD51C, RFWD3, MAD2L2, XRCC2, SNX5, FEN1, SPTAN1, ATM, ZNF276, ZBTB32, CHEK1, BLM, MRE11, PSAP, KITLG, MUS81, HPRT1, NEK2, NBN, LIG4, DKC1, H2AX, LMNA, CASP3, IL3, ERCC2, IRF1, HELLS, TERC, FAAP24, MLH1, FAAP20, USP1, IMMTP1, NDUFA1, TUBG1, VIM, DDX11, RAD18, SLX1A, SLX1B, WDR48, FAAP100, DCLRE1A, PARP1, FADS2, RAD50, BHLHE40, FAN1, FLT3, DCLRE1B, TOP1, REV1, DNA2, NPM1, STAT1, SCD, CENPS, TP53BP1, WRN, TOPBP1, ZBTB16, ATRIP,  UIMC1, THPO, RNF8, DHFR, HELQ, POLN, TP53, RTEL1, ERCC1, ATAD5, EME1 |
| DC | DKC1, TERT, TINF2, RTEL1, PARN, CTC1, TERC, WRAP53, NOP10, NHP2, USB1, ACD, TP53, DCLRE1B, CSF2, PRDM8, SNORA36A, TRUB2, GAR1, TRUB1, CSF3, POT1, LIG4, GRHL2, TNFRSF6B, NUTM1, SNORA56, RMND5B, ARFRP1, PARD6A, PFAS, LINC00324, AP4B1, MAGEA1, MAGEA10, MAGEA11, MAGEA2, MAGEA2B, MAGEA3, MAGEA4, MAGEA5 |
| SDS | SBDS, SRP54, DNAJC21, EFL1, AGO2, L3MBTL1, SRP72, ELANE, GATA2, CSF3R, RPL5, EIF6, NIP7, LGALS9C, SGK2, RPS27L, ARFIP2, RPS14 |
| IBMFs | TERC, TERT, TINF2, TRF1/TRF2, CTC1, SBDS, FANC, NBS1, PRF1, STAT1(GOF), THPO, MPL, SAMD9/9L, GATA2 |
| PNH | PIGA, PIGT, CD59, CD55, C5, FCGR3B, CD14, CD58, PLAUR, HP, PLAU, PLCG1, BST1, GFRA1, LOC110806306, TERC, LOC107985405, MIR6812 |
| DBA | RPS19, RPL5, RPS24, RPL11, RPS10, GATA1, TP53, RPS26, RPS17, RPL26, TSR2, RPL15, RPL35A, RPS29, RPS28, RPS15A, RPS7, RPL35, RPL18, ADA2, RPS27, RPL27, DBA2, EPO, KITLG, IL3, FLVCR1, RPS14, THPO, RPS16, RPS15, RPL36, RSL24D1, RPL31, RPS27A, RPS2, RPL23A, RPL3, KCNN4, RNF168, RPS3A, FLI1, BAMBI, TCF12, MPL, KLF1, CSF2, DIPK1A, NUDT3, LOC105369780, IQCG, MIR6797, SNORD21, EPOR, LOC107987206, POLR3A, RN7SL1, SNORA66, PRL, NDUFA7, NKIRAS1, RAB13, SPHK2, WDR38, COL3A1, SEC23B, CDAN1, C15orf41 |
| CN | CSF3R, JAGN1, HAX1, GFI1, G6PC3, ELANE, CSF3, VPS45, WAS, PTPN11, IL3, KITLG, CSF2, JAK2, BIRC5, LEF1 |

Note：FA, Fanconi anemia; DC, dyskeratosis congenita; SDS, Shwachman-Diamond syndrome; IBMS, inherited bone marrow failure syndrome; DBA, [Diamond-Blackfan anemia](https://pubmed.ncbi.nlm.nih.gov/32309614/); PNH, paroxysmal nocturnal hemoglobinuria; CN, [congenital neutropenias](https://pubmed.ncbi.nlm.nih.gov/28593997/); IT, inherited thrombocytopenia.
